# Protective Role of *Spirulina platensis* against Bifenthrin-Induced Reprotoxicity in Adult Male Mice by Reversing Expression of Altered Histological, Biochemical, and Molecular Markers Including MicroRNAs

**DOI:** 10.3390/biom10050753

**Published:** 2020-05-12

**Authors:** Mohamed Barkallah, Ahlem Ben Slima, Fatma Elleuch, Imen Fendri, Chantal Pichon, Slim Abdelkafi, Patrick Baril

**Affiliations:** 1 Unité de Biotechnologie des Algues, Biological Engineering Department, National Engineering School of Sfax, University of Sfax, 3038 Sfax, Tunisia; mohamedbarkallah@gmail.com (M.B.); fatma.elleuch@ymail.com (F.E.); 2 Faculté des Sciences de Sfax, Université de Sfax, 3029 Sfax, Tunisia; ahlem_benslima@yahoo.fr; 3 Laboratoire de Biotechnologie Végétale Appliquée à l’Amélioration des Cultures, Faculté des Sciences de Sfax, Université de Sfax, 3029 Sfax, Tunisia; imen.fendri@fss.usf.tn; 4 Centre de Biophysique moléculaire (CBM), CNRS UPR 4301, Université d’Orléans, 45071 Orléans, France; pichon@cnrs-orleans.fr (C.P.); patrick.baril@cnrs.fr (P.B.)

**Keywords:** Reprotoxicity, pesticides, oxidative stress, apoptosis, microRNA, *Spirulina* antioxidants

## Abstract

The potential reprotoxicity of bifenthrin remains unclear if only the common clinical indicators of reproductive disease are examined. The present study aimed to investigate the efficacy of *Spirulina platensis*, a microalga rich in antioxidant compounds, against bifenthrin-induced testicular oxidative damage in male mice. At the first, we demonstrate that administration of bifenthrin resulted in a decline of testosterone level and in deterioration of sperm quality that was correlated with significant transcription changes of some specific mRNA and microRNA involved in cholesterol transport, testosterone synthesis, and spermatogenesis. At the biochemical level, we found that oxidative stress was obvious in the bifenthrin group, as evidenced by increase in malondialdehyde (MDA), protein carbonyls (PCO), reactive oxygen species (ROS), and nitrite oxide (NO) that was correlated with activation of genes related to mitochondrial apoptotic signal pathways. We then brought, for the first time to our knowledge, solid and complete experimental evidences that administration of mice with *Spirulina* extract was sufficient to protect against deleterious effects BF in testicular tissues by abrogating the change in antioxidant enzyme activities; the increase in MDA, PCO, and NO concentrations; and the altered expression level of miRNA and mRNA involved in spermatogenesis. We finally demonstrate that *Spirulina* restores the production of testosterone in mice as well as epididymal sperm viability and motility. These results suggest a potential antitoxic activity of Tunisian *Spirulina* deserving further attention.

## 1. Introduction

Humans are frequently exposed to pesticides either directly, as workers in agricultural and industrial environments, or indirectly, via the consumption of food (vegetables, fruits, and cereals) and contaminated water. Many of these toxic chemical products may disrupt human reproduction by interfering with the endocrine function either by mimicking, modulating, or blocking the synthesis and metabolism of reproductive hormones such as estrogen and progesterone in women and testosterone in men.

High levels of pesticide exposure may induce toxicity in testicular tissues, thus disrupting testosterone synthesis and sperm production and/or quality and ultimately could reduce male fertility. Consequently, it is not surprising that the number of scientific studies on the reprotoxicity of pesticides has increased exponentially over the past 20 years, particularly in industrial-agricultural countries [1].

Bifenthrin (BF) (2-methylbiphenyl-3-ylmethyl-(z)-(1RS)-cis-3-(2-chloro-3,3,3-trifluoroprop-1-enyl)-2,2-dimethylcyclopropane carboxylate) is a type I synthetic pyrethroid used extensively against a broad spectrum of insect pests in agriculture and horticulture. As a potential food contaminant, BF can be commonly ingested by humans. Many recent experimental studies have demonstrated that BF is genotoxic, carcinogenic, neurotoxic and exhibits immunosuppressive and reprotoxic effects in a range of mammalian species and even in humans [2,3]. In addition, BF has been reported to induce oxidative stress in human erythrocytes as well as mouse hepatic cells [4] and to induce apoptosis in murine macrophages [3] and human hepatocarcinoma cells. However, scientific knowledge on the mechanism by which BF induces reprotoxicity in in vivo models is currently limited to two main reports [5,6].

Based on these studies, it has been proposed that the toxic effects of BF on reprotoxicity can occur through the production of high levels of mitochondrial reactive oxygen species (ROS), causing oxidative damage to various cellular components [4]. Abnormal ROS production can induce detrimental chemical and structural modifications to sperm nuclear DNA as well as protein damage in sperm plasma and mitochondrial membranes [7]. Mammalian sperm plasma membranes are extremely susceptible to lipid peroxidation induced by ROS because they have a high content of polyunsaturated fatty acids and insufficient antioxidant defense mechanisms [1]. Therefore, the body and specially the testis tissues need additional strategies to defend against excessive ROS production induced by BF exposure. Nowadays, there is an increased demand for using dietary supplements in the prevention and treatment of ROS-related diseases.

Microalgae have attracted attention in the food industry and the principal genera used for functional foods are *Chlorella*, *Dunaliella*, and *Spirulina* [8,9,10]. *Spirulina platensis* (SP) is a unicellular cyanobacterium with a special composition of nutritional and bioactive substances (proteins, vitamins, minerals, pigments and phenolic acids, etc.) that are potential sources for a large range of medical applications [11]. This cyanobacterium contains highly potent naturally antioxidant and free radical scavenging agents such as phycocyanin and beta-carotene [12] that are well-known to protect against various diseases, such as renal failure and cancers [13,14]. Therefore, it is not surprising that SP is generating growing interest in the scientific community because of its remarkable multi-organ protection property against many environmental toxic chemicals and heavy metal–induced toxic assaults [15].

The aims of the present study were to (i) assess the reprotoxicity of BF in adult male mice using multilevel evaluations of testicular tissue including histology, oxidative enzyme activity monitoring and alterations of microRNA (miRNA) and mRNA expression levels and (ii) investigate the possible protective role of SP against the reprotoxicity effect induced by BF in this animal model of pathology. We provide a complete series of solid evidence that SP can be considered as a palliative treatment to protect against infertility in populations heavily exposed to pesticides.

## 2. Materials and Methods

### 2.1. Chemicals and Reagents

SP was isolated, identified and produced as described previously by Ben Amor et al. [11]. All chemicals and reagents were purchased from Sigma-Aldrich Corp. (St. Louis, MO, USA). The testosterone ELISA kit was purchased from BioVendor R&D (Brno, Czech Republic).

### 2.2. Evaluation of Physicochemical, Nutritional and Microbiological Qualities, and Antioxidant Activity of the Isolated SP

The physicochemical characteristics (pH, proteins, lipids, carbohydrates, sugars, fibers, minerals, etc.) and pigment content (chlorophylls, carotenoids, phycocyanins) of the SP dry powder were determined as described by Barkallah et al. [16]. Concentrations of calcium, magnesium, potassium, sodium and iron were measured by atomic absorption spectroscopy (JY 38 S; Horiba, Montpellier, France). The fatty acid methyl esters (FAMEs) of total lipids were obtained by adding 500 µL of KOH (1N)–CH_3_OH (2N) to the extracted lipids followed by a heating step of 10 min at 40 °C and the addition of 500 µL of n-hexane to the reaction mixture. The FAMEs in supernatants were then analyzed using gas chromatography (GC, Shimadzu GC-17A, Shimadzu Scientific Instruments, Columbia, MD, USA) and identified by comparison of their retention times with respect to pure standards of FAMEs purchased from Sigma and analyzed under the same conditions. FAMEs were quantified according to their percentage area, obtained by integration of the peaks. Vitamins were measured using the HPLC system method according to the Association of Official Analytical Chemists [17]. The antioxidant activity of SP was evaluated according to the method described by Bersuder et al. [18] using 1,1-diphenyl-2-picryl-hydrazil (DPPH) as indicator. Total viable bacterial count, mesophilic bacteria, yeasts and molds, and coliform group were enumerated (CFU g^−1^) using the standard microbiological methods for the analysis of food [19]. *Salmonella* spp., *Listeria* spp., and *Staphylococcus aureus* were detected according to the methods established by Barkallah et al. [16]. Metals in SP were determined using inductively coupled plasma-atomic emission spectrometry (JY 38 S; Horiba, Montpellier, France).

### 2.3. Animal Care

Approximately eight-week-old white Swiss male mice weighing 29 ± 3 g were obtained from provided by the Centre of Veterinary Research of Sfax, Tunisia. Experimental animal procedures were performed according to the recommendations of the European convention for the protection of vertebrate animals and in accordance with the Council Directive no. 2010/63/EU. Animals were kept in an air-conditioned room (22 ± 3 °C) with a relative humidity of approximately 40% and housed in stainless steel cages and subjected to a normal photoperiod (12 h dark/12 h light) before the beginning of the experiment. They were allowed free access to diet and water for one week during their acclimatization period and were daily observed to detect any source of suffering or abnormal behavior.

### 2.4. Experimental Protocol

After one week of acclimatization, the mice were randomly divided into four different groups of eight animals each. The first group of animals was administered physiological saline buffer (0.9% salt solution) and was used as a negative control group (C). The second group (BF) of animals was administered by oral gavage with BF at a dose of 5 mg/kg body weight on a daily basis during a period of 35 days. The third group (SP) of animals was also administered by oral gavage with SP but at a dose of 500 mg/kg on a daily basis over 35 days. The fourth group (SP + BF) of animals was administered with SP at a dose of 500 mg/kg/day, 2 h before BF administration at the same dose regimen as the second group of animals. These doses of BF and SP were selected based on our preliminary experiments (data not shown).

At the end of the treatment period, the animals of the different groups were weighed and sacrificed by cervical decapitation to avoid animal stress. The collected blood samples were left to clot at room temperature and then centrifuged at 3000 rpm for 15 min. Sera were then collected and stored at −20 °C for further biochemical analysis. The testes and epididymis were quickly excised from each animal, rinsed in ice-cold physiological saline buffer, and weighed to calculate the ratio of the organ weight to the body weight (%). Representative samples of testes were collected and fixed in 10% formalin solution for histological analysis. Other testes samples and the tails of the epididymis were used immediately for the analysis of different molecular and biochemical parameters and to study sperm parameters.

### 2.5. Sperm Collection and Analysis

Epididymal spermatozoa were collected by cutting the caudal region of the right epididymis into small pieces of approximately 5 mm and then incubated in 2 mL of pre-warmed physiological buffered saline (PBS) at 37 °C for 10 min to allow sperms to swim out. After a centrifugation step at 1600× *g* for 15 min, supernatants were collected to evaluate the cell concentration, motility, viability and morphology of sperms by histological examinations. Briefly, sperm motility was analyzed microscopically by determining the number of all progressive spermatozoa from the total spermatozoa population. The final data were expressed as the percentage of sperm cells in each motility group as previously described by Kvist and Björndahl [20]. 

Sperm viability was defined as the percentage of normal cells, according to the procedure described in the World Health Organization Manual [21]. The study was assessed using the one-step eosin-nigrosin staining technique.

To evaluate the various abnormalities of spermatozoa, sperm suspension was stained with 0.2% final volume of eosin on slides before histological classification as previously described by Wyrobek and Bruce [22].

### 2.6. Determination of Oxidative Stress Markers

Lipid peroxidation in the testicular tissue was estimated by measurement of malondialdehyde (MDA) content [23]. Protein carbonyl contents were measured by using the spectrophotometric method [24]. Testicular reactive oxygen species (ROS) levels were measured using DCF-DA (2′,7′-dichlorofluorescein diacetate) as fluorescent probe [25]. The results were expressed as mean fluorescence intensity compared with values of the control group normalized to 100%. Nitric oxide production was determined based on the Griess reaction [26]. The results were expressed as nmol/ 100 mg protein. 

### 2.7. Determination of Non-enzymatic and Enzymatic Antioxidants in Testicular Tissues

The reduced glutathione (GSH) levels in testicular tissues were determined with Ellman’s reagent (DTNB; 5, 5-dithiobis-2-nitrobenzoic acid) as probe to quantify the total GSH content measured at 412 nm [27]. The result was then expressed as µg mg^−1^ of tissue. Catalase (CAT) activity was assayed by the decomposition of hydrogen peroxide (H_2_O_2_) [28]. The enzyme activity was calculated as µmol H_2_O_2_ consumed/min/mg protein. Total superoxide dismutase (SOD) activity was assayed by measuring its ability to inhibit the photochemical reduction of nitroblue tetrazolium (NBT) [29]. Results were then expressed as enzyme unit activity mg^−1^ protein. Glutathione peroxidase activity (GPx) was measured as previously described by Flohe and Gunzler [30]. The final results were then expressed in terms of nmol GSH oxidized/min/mg protein.

### 2.8. Estimation of Testosterone in Sera

The testosterone level in mice sera was quantified using the Mouse Rat Testosterone ELISA Kit (BioVendor, Asheville, NC, USA) according to the manufacturer’s instructions.

### 2.9. Analysis of DNA Fragmentation

Testicular DNA samples of normal and experimental mice were isolated as previously described [31].

### 2.10. Analysis of Gene Expression by Quantitative RT-PCR (qRT-PCR)

Extraction of total RNA from frozen testicular tissues was performed using the mirVana microRNA isolation kit with phenol according to the manufacturer’s instructions (Thermo Fisher Scientific, Inc., Waltham, MA, USA). RNA integrity was assessed with the BioAnalyzer 2100 (Agilent technologies, Santa Clara, CA, USA). Samples with an RNA integrity number (RIN) superior or equal to 8 were considered for further analysis. The reverse transcription step was performed with the PrimeScript RT reagent Kit with gDNA eraser kit for the mRNA analysis while the TaqMan^®^ Advanced miRNA cDNA Synthesis Kit was used for the miRNA analysis (Applied Biosystems company, Thermo Fisher Scientific, Inc., Waltham, MA, USA). PCR products were generated from 100 ng of cDNA template using the QuantiFast SYBR Green PCR Master mix (Qiagen, Germantown, MD, USA) with specific forward and reverse primers to detect the expression of mRNAs or with a mixture of specific forward primers complementary to each miRNA mature sequence of interest used in combination with the universal qPCR reverse primer provided by the NCode VILO miRNA cDNA synthesis kit (Thermo Fisher Scientific, Inc., Waltham, MA, USA). The PCR primers used in this study are shown in Table S1. All reactions were performed in triplicate on a Lightcycler^®^ 480 Instrument II (Roche, Basel, Switzerland). Optimal q-PCR parameters were one cycle of 95 °C for 2 min followed by 40 cycles of 15 s at 95 °C and 1 min at 60 °C. A melting curve analysis was performed using the following cycling parameters: 60 °C for 30 s and 5 °C temperature changes to the end temperature of 95 °C. For all samples, the mRNA expression level was normalized to the housekeeping beta-actin gene and to the snU6 level for quantification of mature miRNA. Finally, the relative expression levels of mRNA and miRNA were calculated using the standard 2^−ΔΔCt^ method.

### 2.11. Protein Quantification

Total protein concentration was measured using pure bovine serum albumin (BSA) as standard [32].

### 2.12. Testicular Histopathology

For the histological study, mice testes were removed and fixed in 10% buffered formalin. After routine paraffin processing, embedded testicular tissue samples were sectioned at 5 mm thickness using a Reichter 2040 Microtome (Medical Equipment Source, LLC; PA, USA) and stained with hematoxylin and eosin (H-E). The prepared sections were examined with a Leica^®^ microscope fitted with a Sony^®^ digital camera to capture images for the histological evaluation of testicular tissue alterations.

### 2.13. Statistical Analysis

All analytical determinations were performed at least in triplicate and values were expressed as the mean ± standard error of mean (SEM). One-way ANOVA and Tukey’s post-hoc multiple comparison tests were used to compare results with significant differences (*p* < 0.05). GraphPad Prism 6.0 for Windows (Graph Pad Software, San Diego, CA, USA) was used to perform all statistical analyses.

## 3. Results

### 3.1. SP as a Source of Nutritional and Bioactive Compounds

A set of quantitative analyses was conducted to provide information regarding the composition of SP in terms of its nutritional and bioactive compounds. Table 1 shows the results of the physicochemical and microbiological assessment on SP powder. This study shows that dried SP is principally composed of proteins and carbohydrates, which account for 64.35% and 21.9% of SP dry weight respectively while lipids and minerals account for 7.46% and 6.8%, respectively, in the remaining biomass. The SP fatty acid composition determined by GC-FID analysis is displayed in Table 1. SP was found to contain substantial proportion of PUFAs and MUFAs, which account for 41.5% and 14.78%, respectively, of the total fatty acid methyl esters (FAMEs) content.

As a precursor of unsaturated fatty acids, palmitic acid is present and was quantified as 39% of the total FAMEs. SP was also found to be rich in gamma-linolenic acid (GLA) (22% of the total FAMEs) and to contain high levels of omega 3 (*ω3*), omega 6 (*ω6*) and omega 9 (*ω9*), which account for 6.53%, 18.02%, and 8.25%, respectively, of the total FAMEs present in the sample.

Furthermore, SP contains essential minerals such as calcium (998 mg/100 g dw), magnesium (1.35 mg/100 g dw), potassium (2150 mg/100 g dw), sodium (1380 mg/100 g dw), and iron (336 mg/100 g dw) (Table 1). Dry SP is also rich in B-group vitamins (B1 (5.53 mg/100 g dw), B2 (4.99 mg/100 g dw), B7 (46 mg/100 g dw) and B9 (9.88 mg/100 g dw), and vitamin E (8.98 mg/100 g dw) (Table 1).

At an extract concentration of 100 µg mL^−1^, SP showed a DPPH scavenging activity of 42% (Table 1). Furthermore, chlorophylls, beta-carotene, and phycocyanin were found as principal pigments in SP. They accounted for 2350, 1480, and 52 mg/100 g of dry SP, respectively (Table 1).

No traces of mold, yeast or foodborne pathogens (*Escherichia coli*, *Salmonella* spp., *Listeria* spp., and *Staphylococcus aureus*) were detected in SP samples during two months of storage at 4 °C. Dry SP was also tested and found to be free of pesticides (Table 1).

All these nutritional and hygienic properties make this product a valuable element for testing its therapeutic effects against BF-induced infertility in male mice.

### 3.2. Effect of BF and/or SP Treatment on General Health, Body Weight and Reproductive Organs Weight

The results presented in Table 2 show that BF administration significantly decreased the body weight gain by seven-fold (*p* < 0.01) and testicular weight by 0.43-fold (*p* < 0.01) in BF-exposed mice compared to control mice. Remarkably, the administration of SP prior to BF normalized the body weight gain (*p* < 0.01). The weight of the testes was close to that of the control group in comparison with the BF group. No significant changes were observed in epididymal weight in all groups of animals (*p* > 0.05) (Table 2).

### 3.3. Effect of BF and/or SP Treatment on Seminal Picture

**Figure 1:**
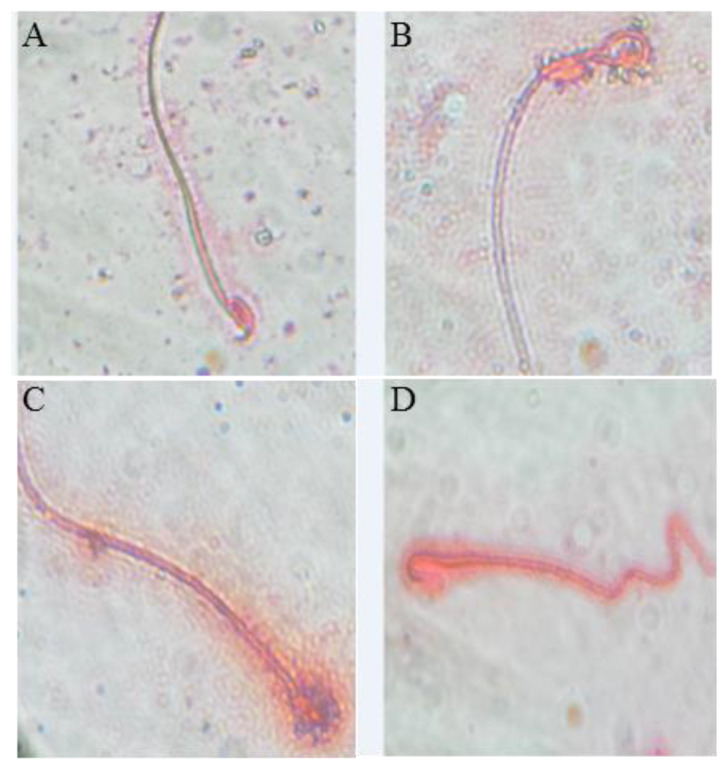
Observation under an optical microscope of the eosin-stained spermatozoa of mice (100 × magnification), (**A**) control (normal morphology of the spermatozoa); (**B**) mid-piece abnormality of the spermatozoa; (**C**) head abnormality of the spermatozoa; (**D**) tail abnormality of the spermatozoa.

As shown in Figure 1, the counts of epididymal sperm, viability and motility percentages were significantly decreased by 61%, 3%, and 41.3%, respectively in the BF-exposed group. Also, a twofold significant increase (*p* < 0.01; n = 3) in sperm abnormalities was observed in the BF treated mice including mid-piece anomalies (75%) (Figure 1B), head (75%) (Figure 1C), and tail anomalies (66%) (Figure 1D) when compared to the control group (*p* < 0.01) (Figure 1A).

These anomalies were counted in each group and are classified according to their intensities in Table 3. Interestingly, the administration of SP prior to BF resulted in significant increases (*p* < 0.01; n = 3) in sperm count (+ 136%), viability and motility percentages (+80%) compared to the BF group of mice (Table 3). The percentage of spermatozoa with abnormal morphology was significantly reduced (*p* < 0.01, n = 3) by 42% in the group treated with the combination of SP + BF as compared to the BF group (Table 3).

### 3.4. Histological Change in Mouse Testes

**Figure 2:**
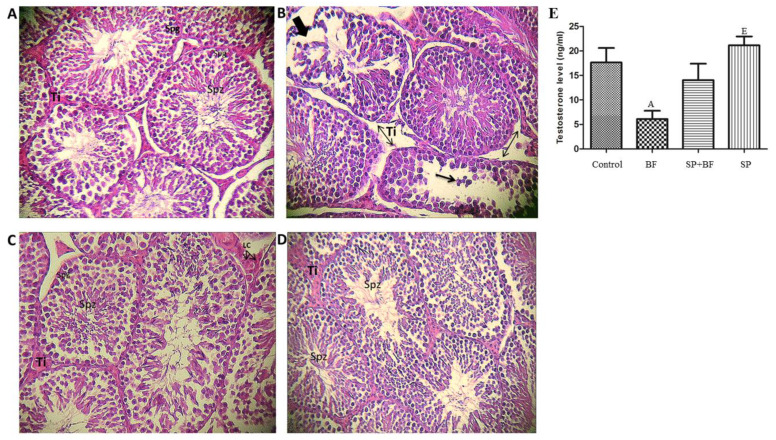
(**A**) Testicular sections of control mice, which show normal spermatogenesis (400× haematoxylin and eosin (H&E)): note the normal cell arrangement in the seminiferous tubules. The interstitial spaces also appear normal. (**B**) Testicular sections of mice treated with 5 mg per kg b.w. per day of BF (400 × H&E): note the atrophic seminiferous tubules with a large proportion of tubules showing signs of degeneration and disorganization. Sloughing of germ cells into tubular lumen. (**C**) Testicular sections of mice treated with 5 mg per kg per day of BF and 500 mg per kg per day of SP (400 × H&E): note the increase of germ cells in the seminiferous tubules. The interstitial spaces appear normal. (**D**) Testicular sections of mice treated with 500 mg per kg per day of SP (400 × H&E): note the normal cell arrangement in the seminiferous tubules. The interstitial spaces also appear normal. Ti, interstitium; Sg, spermatogonia; Spzs, spermatozoa. (**E**) Effect of different treatments on testosterone level. Values are expressed as means SD of eight mice in each group. All groups *vs.* control group: ^A^ *p* < 0.05; ^B^ *p* < 0.01; ^C^ *p* < 0.001. All groups *vs.* BF group: ^D^ *p* < 0.05; ^E^ *p* < 0.01, ^F^ *p* < 0.001.

The testes of controls (Figure 2A) and mice administered with SP (Figure 2D) showed normal morphology with dynamic spermatogenesis. In fact, seminiferous tubules presented a complete spermatogenesis and a high density in spermatozoa inside the lumen of the seminiferous tubules. In sharp contrast, BF caused severe damage to the seminiferous tubules with apparent disordered and hollow structures.

The atrophic seminiferous epithelium showed a large proportion of tubules with signs of degeneration and disorganization. Disarray and desquamation of the spermatogenic cells were also observed (Figure 2B). Remarkably, the administration of SP prior to BF induced a significant improvement in histopathological observation (Figure 2C). In addition, the testicular structure in this group was almost completely restored to normal, with an active spermatogenesis, well-preserved tubular morphology, and active sperms in the lumen of seminiferous tubules (Figure 2C).

### 3.5. Effect of BF and/or SP Treatment on Plasma Testosterone

We then assessed the plasma level of testosterone in mice and found a statistically significant decrease (-61%; *p* < 0.05; n = 8) of testosterone in the 5 mg/kg BF-treated mice when compared with the control group of mice (Figure 2E). The administration of SP prior to BF remarkably increased the testosterone level (+110%; n = 8) when compared with the BF group. In contrast, no significant difference was observed in mice treated with SP alone when compared with the control group.

### 3.6. Effect of SP on Testicular Parameters of BF Intoxicated Male Mice

#### 3.6.1. ROS Level and Oxidative Stress Markers (MDA, PCO, NO)

**Figure 3:**
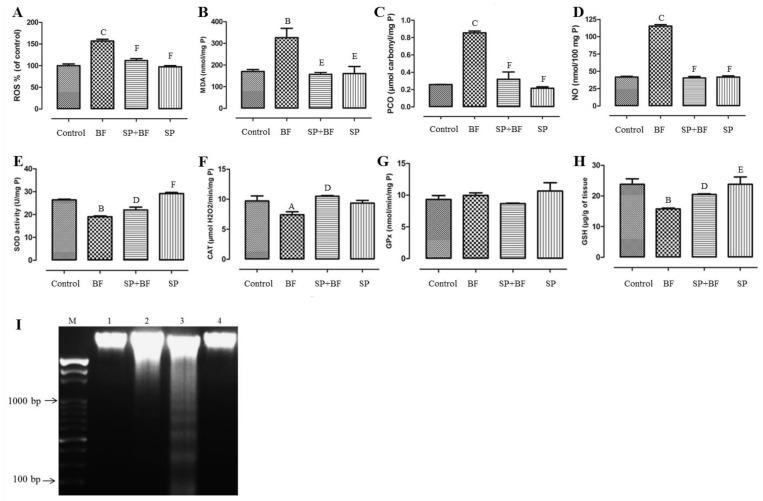
Effect of different treatments on ROS (**A**), MDA (**B**), PCO (**C**), NO (**D**), levels, antioxidant enzyme activities (SOD (**E**), CAT (**F**), and GPx (**G**)), GSH concentration (**H**) and DNA fragmentation (**I**) in testes of controls and mice treated with bifenthrin (BF), Spirulina (SP), or their combination (SP + BF). Values are expressed as means SD of eight mice in each group. All groups *vs.* control group: ^A^ *p* < 0.05; ^B^ *p* < 0.01; ^C^ *p* < 0.001. All groups *vs.* BF group: ^D^ *p* < 0.05; ^E^ *p* < 0.01, ^F^ *p* < 0.001.

As shown in Figure 3A, BF significantly increased the level of ROS (+60%; *p* < 0.001; n = 8) in comparison with the control group. The administration of SP prior to BF resulted in a significant decrease (−29%; *p* < 0.001; n = 8) in ROS levels in comparison with the BF group (Figure 3A). Likewise, BF administration for 35 days induced a significant, approximately 1.8-fold increase (*p* < 0.01, n = 8) in the production of MDA compared to the control group (Figure 3B). The administration of SP prior to BF significantly reduced the increased level of MDA about twofold (*p* < 0.01; n = 8) in comparison to the control group (*p* > 0.05). Furthermore, BF also increased the level of PCO by four in comparison with the control group (*p* < 0.001; n = 8). The administration of SP prior to BF reduced the increased level of PCO about 3.7-fold (*p* < 0.001; n = 8) in comparison with the BF group (Figure 3C). Compared to control mice, an approximately threefold increase in NO level (*p* < 0.001; n = 8) was recorded in the testes of mice treated with BF (Figure 3D). On the other hand, mice treated with SP prior to BF exhibited a significant suppression in the level of NO (three-fold; *p* < 0.001; n = 8) compared to mice that received BF alone.

#### 3.6.2. Enzymatic and Non-Enzymatic Antioxidants

Figure 3 illustrates the level of antioxidant enzymes and indicates that mice exposed to BF showed a remarkable reduction in testicular SOD (Figure 3E) and CAT (Figure 3F) activities by 28% and 25% respectively, relative to the control group. On the other hand, no significant difference was observed in testicular GPx activity in all groups of animals (*p* > 0.05; n = 8) (Figure 3G). In addition, a 35% depletion in the GSH pool, compared to the control group, was recorded following BF administration (*p* < 0.01; n = 8) (Figure 3H). Remarkably, yet again, the administration of SP prior to BF significantly increased the enzymatic antioxidant activities (*p* < 0.05; n = 8) and GSH content (*p* < 0.05; n = 8) when compared with the BF group. It is worth noting that no difference was observed in mice treated with SP alone when compared with the control group (*p* > 0.05; n = 8).

#### 3.6.3. Effect of SP on Oxidative DNA Fragmentation Induced by BF

DNA fragmentation was analyzed using agarose gel electrophoresis to visualize the possible presence of a DNA fragmentation pattern around 150–200 bp. The results of this analysis are given in Figure 3I. The testicular DNA was found intact in the control (Lane 1) and the SP-treated animals (Lane 4), whereas BF-treated animals showed a streak of marked DNA fragmentation in the testis tissue when compared to the controls (Lane 3). Remarkably, mice treated with SP prior to BF showed an almost total reversibility effect of DNA fragmentation. No DNA fragmentation was detected in this group of animals (Lane 2).

#### 3.6.4. Changes in the Transcription of Genes Related to Cholesterol Transport and Testosterone Synthesis

**Figure 4:**
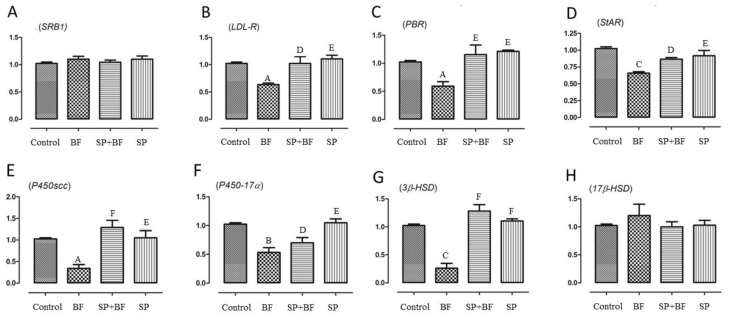
Effects of different treatments on mRNA levels of genes related to cholesterol transport and testosterone synthesis including SRB1 (**A**), LDL-R (**B**), PBR (**C**), StAR (**D**), P450scc (**E**), P450-17α (**F**), 3β-HSD (**G**) and 17β-HSD (**H**) in testes of mice. Values are presented as means ± SE. All groups *vs.* control group: ^A^ *p* < 0.05; ^B^ *p* < 0.01; ^C^ *p* < 0.001. All groups vs. BF group: ^D^ *p* < 0.05; ^E^ *p* < 0.01, ^F^ *p* < 0.001.

To test whether BF exposure would affect cholesterol transport in the testis, we examined the mRNA expression levels of SRB1, LDL-R, PBR, and StAR. It is known that SRB1 and LDL-R are responsible for the transport of cholesterol from blood to testis while PBR and StAR play key regulatory roles in cholesterol transport from the outer to the inner mitochondrial membrane. As shown in Figure 4B–D, expressions of LDL-R, PBR, and StAR were significantly down-regulated by 36% (*p* < 0.01; n = 6), 41% (*p* < 0.01; n = 6), and 35% (*p* < 0.01; n = 6) in the BF-treated group of animals when compared to the control group. Again, and interestingly, the administration of SP prior to BF reversed the relative mRNA expression levels of LDL-R, PBR, and StaR to the control expression level (*p* < 0.01) (Figure 4B–D). No significant difference was observed in the expression level of SRB1 in any of the groups of animals (Figure 4A).

Next, we investigated the expression level of key genes implicated in the testosterone synthetic pathway, including *P450scc*, *3β-HSD*, *P45017α*, and *17β-HSD*. Exposure of mice to BF significantly reduced the mRNA levels of P450scc by 73.5% (Figure 4E), P450-17α by 45% (Figure 4F), and 3β-HSD by 76% (Figure 4G) when compared to the control group of animals, while it did not cause any significant difference in the expression of 17β-HSD (*p* > 0.05; n = 6) (Figure 4H). 

Again, and remarkably, the expression of P450scc, P450-17α and 3β-HSD mRNAs was partially to almost totally reversed to the expression level detected in mice treated first with SP before administration of BF (*p* < 0.001) (Figure 4E–G). No significant difference was observed in the mRNA levels of these genes in the testes of SP and control groups of mice.

#### 3.6.5. Changes in the Transcription of Genes on the Apoptosis-Inducing Pathway

**Figure 5:**
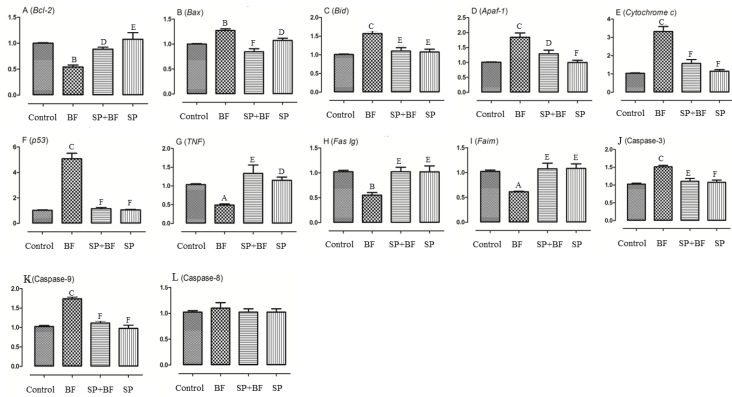
Assessment of apoptosis-related genes in mice testes. The quantifications of apoptosis related genes including Bcl-2 (**A**), Bax (**B**), Bid (**C**), Apaf-1 (**D**), Cytochrome c (**E**), p53 (**F**), TNF (**G**), Fas lg (**H**), Faim (**I**) and caspases 3 (**J**), 9 (**K**), 8 (**L**) was performed by real-time quantitative polymerase chain reaction (RT-PCR). All samples were run in triplicate and results are presented as mean ± SEM. All groups *vs.* control group: ^A^ *p* < 0.05; ^B^ *p* < 0.01; ^C^ *p* < 0.001. All groups *vs.* BF group: ^D^ *p* < 0.05; ^E^ *p* < 0.01, ^F^ *p* < 0.001.

We then investigated the apoptotic status of testes tissues treated with BF and compared it with the group of animals initially treated with SP. As Bcl-2 family members are a central regulator of caspase activation, the expression of these apoptosis markers was investigated and compared between each group of animals. As shown in Figure 5A, Bcl2 mRNA was found to be down-regulated by 45% (*p* < 0.05) in the 5 mg/kg BF treatment group (Figure 5A) when the expression of the pro-apoptotic genes, Bax, was found to be up-regulated by 27% (*p* < 0.01; n = 6) (Figure 5B,C). As a result, the Bcl-2/Bax ratio, a major checkpoint in the apoptosis pathway, was significantly lowered. Additionally, Bid mRNA was up-regulated by 2.72-fold (*p* < 0.01) in the 5 mg/kg BF treatment group (Figure 5C). Remarkably, the administration of SP prior to BF partially restored all of these altered markers of testes apoptosis to the control expression level. The ratio of Bcl2 and Bax mRNA expression (Bcl2/Bax ratio) was significantly increased by 2.72- fold in the SP+BF group (*p* < 0.001) compared to the BF treatment group, i.e., close to the ratio detected in the control group of mice. No significant difference was observed in mRNA levels of these genes in the testes between the SP group and controls.

Cytochrome c and apoptosis protease-activating factor 1 (Apaf-1) were then evaluated to determine whether BF-induced testicular apoptosis was related to a change in mitochondrial expression. As shown in Figure 5E, cytochrome c was significantly up-regulated in the 5 mg/kg BF treatment group showing a 2.8-fold increase (*p* < 0.05) and Apaf-1 expression level was also increased by 1.9-fold (*p* < 0.05) in the 5 mg/kg BF treatment group (Figure 5D).

Additionally, the mRNA levels of caspase 3 and caspase 9 were up-regulated by 1.5 and 1.75-fold respectively, in the 5 mg/kg BF treatment group (Figure 5J,K). However, no significant difference in the expression of caspase 8 was found between the different groups of animals (*p* > 0.05; n = 6) (Figure 5L). Similarly, the expression of p53 was significantly increased five-fold (*p* < 0.01) in the 5 mg/kg BF treatment group (Figure 5F). Remarkably, the administration of SP prior to BF significantly reversed the relative mRNA expression level of cytochrome c, Apaf-1, and p53 in the testis when compared with the BF group (p< 0.01). We then finally investigated whether the tumor necrosis factor (TNF) pathway might be part of the toxicity effect of BF. Our results in Figure 5 indicated that TNF, Fas Ig and Faim were significantly down-regulated by 59.8%, to 56.6% and 58.2% respectively in the 5 mg/kg BF treatment group compared to the control group of mice (Figure 5G–I).

Overall, these results suggest that, at the testicular level, BF triggers the intrinsic mitochondrial apoptotic signals rather than the extrinsic death receptor pathway. Again, the relative mRNA expression levels of TNF, Fas Ig, and Faim were partially to almost totally reversed to the control expression level when the mice were treated first with SP before the administration of BF (*p* < 0.001) (Figure 5G–I).

#### 3.6.6. Changes in the Spermatogenesis and Apoptosis Related miRNA

**Figure 6:**
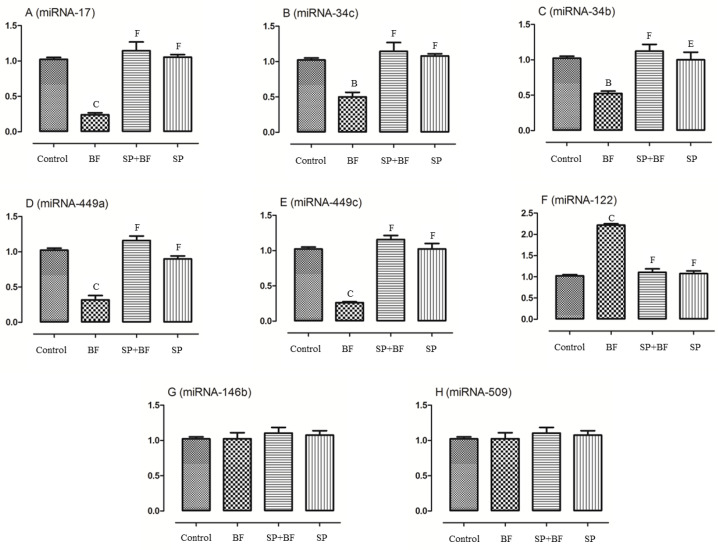
Effects of different treatments on spermatogenesis and apoptosis related miRNA including miR-17 (**A**), miR-34c (**B**), miR-34b (**C**), miR-449a (**D**), miR-449c (**E**), miR-122 (**F**), miR-146b (**G**) and miR-509 (**H**). Values are presented as means ± SEM. All groups vs. control group: ^A^ *p* < 0.05; ^B^ *p* < 0.01; ^C^ *p* < 0.001. All groups *vs.* BF group: ^D^ *p* < 0.05; ^E^ *p* < 0.01, ^F^ *p* < 0.001.

As recent evidence has indicated that miRNA are implicated in the control of spermatogenesis and apoptosis of testes tissues, we evaluated the expression levels of some targeted miRNAs and compared their expression levels among all groups of animals. As shown in Figure 6, the exposure to BF significantly (*p* < 0.001; n = 6) reduced the expression of miRNA-17, miRNA-34c, miRNA-34b, miRNA-449a, and miRNA-449c by 75%, 50%, 50%, 68%, and 75%, respectively (Figure 6A–E) compared to the control group of mice while the expression of miRNA-122 was found to be increased by 120% (*p* < 0.001; n = 6) (Figure 6F). No significant difference in the expression of miRNA-146b and miRNA-509 was found between the different groups of animals (*p* > 0.05; n = 6) (Figure 6G,H). Moreover, and again, the administration of SP prior to BF significantly counteracted the deregulated relative expression level of these testicular miRNAs when compared with the BF group (*p* < 0.001; n = 6) and control group (*p* > 0.05; n = 6) (Figure 6). No significant difference was observed in the expression levels of these miRNAs in the testes tissues of SP and control groups of animals (*p* > 0.05; n = 6) (Figure 6).

## 4. Discussion

This study aimed to investigate the reproductive toxicity effect of BF in adult male mice using a multi-scale level of evaluation, starting from histological analysis of testicular tissues, biochemical measurement of oxidative stress and apoptosis markers to the quantification of the expression of targeted genes and miRNAs known to participate in spermatogenesis. The objective was to collect sufficient robust experimental evidence to gain insight into the underlying mechanisms of testicular toxicity induced by this pesticide. We hypothesized that the administration of SP extract, a microalga previously described as beneficial for other pathologies [13], might also protect mice from the testicular toxicity induced by BF. The results presented here provide, for the first time to our knowledge, solid experimental evidence that the compounds in SP extract exercise a pleiotropic of defense mechanisms by almost completely reversing all the altered markers of testicular toxicity induced by BF. Regarding the urgent need for palliative treatments against the toxic effects of pesticides in general, overall, our study highlights the potential of SP extract as a palliative treatment to treat male fertility in countries exposed to BF.

We first found that the administration of BF for 35 days at a dose of 5 mg/kg bw significantly decreased the body weight of the animals with a pronounced effect on the weight of reproductive organs (testes and epididymis). We demonstrated that the reduction in testicular mass is correlated with a drastic decrease in epididymal sperm count, vitality, and motility, while the histological analysis of tissues revealed degeneration of the seminiferous epithelium, deterioration of germ cells, and shrinkage of seminiferous tubules.

The mechanism by which BF induces such drastic testes alterations is partially known and understood. Only two reports, both from the same group, have investigated this point—first in male offspring [5] and second in adult mice [6]. Results from these studies indicate that BF exposure leads to the overproduction of ROS, oxidative DNA damage, apoptosis of testicular cells, and change in intra-testicular testosterone production. 

In our study, we confirm that BF exercises pleiotropic deleterious effects in testicular tissues, causing a reduction in sperm production. It is reasonable to speculate that lipid peroxidation as well as ROS induction in response to BF might be the primary source of tissue damage as reported elsewhere in other organs [4]. In the testes, excessive ROS can have serious deleterious effects on many cellular components by reacting with polyunsaturated fatty acids to form lipid peroxides [33]. Accumulation of the end products of lipid peroxidation may contribute to a decrease in testicular tissue integrity resulting in a loss of testes weight, apoptosis [4], DNA fragmentation [34], and reduction in the production of testicular androgens as evidenced in our study. We demonstrated that MDA, PCO, and NO levels in the testis were significantly increased in BF intoxicated mice, which might also contribute to testes alteration. We also observed depleted contents of GSH and down-regulated enzymatic activities of the redox system (SOD and CAT) in the testis, which reflects the inability of the antioxidant defense to manage the high levels of ROS produced by BF exposure. Consequently, the overproduction of ROS was accompanied by a significant change in the expression of mitochondrial apoptosis-related genes, p53 gene, and subsequent DNA fragmentation [34]. These data are in line with those of other studies that have reported a change in p53 expression in murine organs after different synthetic pyrethroid treatments [35]. We furthermore observed a drastic change in the transcription status of key genes related to testosterone synthesis such as *P450scc*, *3β-HSD* and *P45017α*. Taken all together, these alterations explain well the reduction in sex hormones production, particularly testosterone, and decrease in sperm production, viability and motility that are the main causes of BF-induced infertility.

We then pursued this analysis further and investigated the hypothesis that BF might also alter the expression of specific miRNAs known to be crucial for spermatogenesis [36]. We focused our attention on some miRNAs identified as biomarkers of male infertility such as miRNA-17, miRNA-34c, miRNA-34b, miRNA-449a and miRNA-449c [36]. We found that the expression of miRNA-17, -34c, -34b, -449a, and miRNA-449c was significantly downregulated in BF-treated animals, while the expression of miRNA-122 was upregulated. Our results are in agreement with those of Hayashi and collaborators [37], who showed that the disruption of miRNA-17 in the testes of adult mice resulted in severe testicular atrophy, empty seminiferous tubules, and depressed sperm production. This miRNA is also required for mouse primordial germ cell development and spermatogenesis [36]. The observed downregulation of miRNA34b/c and miRNA-449 in the BF-treated mice is also in accordance with the results of Wu et al. [36] who reported that reductions in these two miRNAs are causally associated with reduced fertility. Furthermore, Yuan et al. [38] reported that miRNA-34bc/449-deficiency impairs both meiosis and the final stages of spermatozoa maturation. In contrast, the over-expression of miR-122a is reported [39] to induce mitotic arrest and apoptosis of spermatocytes, which is also in agreement with the histopathology results of this study. Therefore, our study indicates that the deregulation in the expression of miRNAs might contribute as a consequence or a cause to the reproductive toxicity effect of bifenthrin in male mice. This deserves deeper investigation, notably to establish their diagnosis values.

We then investigated whether SP, known to exercise a beneficial effect in other pathologies [13], might also provide positive outcomes as a protective and anti-infertility agent. The palliative effects of SP on body weight gain could be due to the fortification of the body by important nutrients such as proteins, vitamins, minerals and amino acids brought by SP. Interestingly, the administration of SP prior to BF significantly retrieved the testosterone level and the expression levels of genes related to cholesterol transport and testosterone synthesis. Our findings are in agreement with those of other authors, who showed that SP intake can effectively recover cadmium- and streptozotocin-induced deregulation of gene expression in the early and final steps of the steroidogenic pathway [40]. The pre-treatment of mice with SP positively improved sperm quality parameters, as manifested by an increase in sperm motility and count. This improvement may be due to the richness of SP in zinc, which improves the activity of alkaline phosphatase enzyme in sperm [41]. The pathological lesions induced by BF were also remarkably reduced by the pre-treatment with SP. The role of SP in modulating these testicular alterations may account for the antioxidant vitamin compounds of SP such as α-tochopherol (vitamin E) or ascorbic acid (vitamin C), which might improve testicular structure and function. Vitamin E could inhibit the peroxidation of cell membrane lipids by trapping lipid peroxyl (LOO^−^) and many other radicals to help in counteracting oxidative damage and maintaining the levels of GSH and ascorbic acid in damaged tissues [42]. The SP antioxidant effect has been linked to other bioactive molecules such as C-phycocyanin, β-carotene and chlorophylls [12,43]. These pigments exert their antioxidant activities by increasing the endogenous antioxidant enzymes and scavenging a variety of reactive species such as superoxide and hydrogen peroxide radicals [15,44]. SP also contains selenium, which is involved in the formation of glutathione peroxidase and other compounds such as selenocycteine and selenoglutathione that are known to counteract the toxic effects of pyrethroids [45]. The ameliorative effect of SP was also accompanied by anti-inflammatory activity attested by a reduction in the expression of TNF-α and the production of NO in the testicular tissues of intoxicated mice. Polysaccharides of SP could also inhibit the expression of TNF-α, IL-1β, and COX-2 [46], as we demonstrated in our study. A similar anti-inflammatory activity of SP was previously documented [47,48]. Furthermore, SP counteracted the BF induced apoptotic events by attenuating the expression of the pro-apoptotic genes and ROS scavenging. A similar antiapoptotic effect of SP was previously documented [49]. SP also regulated the expression of spermatogenesis and apoptosis miRNAs. This finding is in agreement with the results of Śmieszek and collaborators [50], who showed that SP influenced the expression of apoptosis-related miRNAs and mRNA in Caco-2 cancer cells line.

## 5. Conclusions

To sum up, the present study shows that BF intoxication induces lipid peroxidation in testicular tissue, disturbs the antioxidant machinery, and alters the morphology and function of testicular tissues. The administration of SP prior to BF, remarkably, attenuates the toxicity of this pyrethroid in the reproductive system in male mice by almost totally reversing the deleterious effects of BF. Based on the strong experimental evidence reported in this study, we propose that nutritional supplementation with SP during BF exposure, might act as a protective agent against inflammation and oxidative stress, a proposal that deserves further preclinical studies and clinical evaluation in humans.

## Figures and Tables

**Table 1:** Mean values of physicochemical and microbiological qualities, pigments content, and antioxidant activity (1,1-diphenyl-2-picryl-hydrazil (DPPH) assay) of *Spirulina platensis* (SP) dry powder.

Composition	Values	Physical Properties	Values
**Protein (%)**	64.35 ± 1.2	**Appearance**	Uniform powder
**Fat (%)**	7.46 ± 0.42	**color**	Blue green
**Saturated acids**	42.5 ± 1.5	**Odor and taste**	Mild like algae
Lauric (C12:0)	0.9 ± 0.1	**Consistency**	Powder
Myristic (C14:0)	0.53 ± 0.04	**Particles size**	60 mesh (> 98%)
Palmitic (16:0)	39.22 ± 0.8	**pH**	6.9 ± 0.2
Stearic (C18:0)	1.85 ± 0.2		
**Monounsaturated**	14.78 ± 0.22		
Palmitoleic (C16:1)	6.53 ± 0.3		
Oleic (C18:1)	8.25 ± 0.4		
**Polyunsaturated**	41.55 ± 0.78		
Linoleic (C18:2)	18.02 ± 0.24		
Gamma-linolenic (C18:3)	22.27 ± 0.1		
Dihomo-γ-linolenic (C20:3)	1.26 ± 0.08		
**Carbohydrate (%)**	21.9 ± 1.1		
**Total dietary fiber (%)**	8.85 ± 0.36		
**Sugar (%)**	2.9 ± 0.5		
**Starch**	3.7 ± 0.1		
**Ash (%)**	6.8 ± 0.05		
Iron (mg/100 g on dry weight basis)	336 ± 19.8		
Calcium (mg/100 g on dry weight basis)	998 ± 14.5		
Magnesium (mg/100 g on dry weight basis)	1.35 ± 0.03		
Potassium (mg/100 g on dry weight basis)	2150 ± 37.5		
Sodium (mg/100 g on dry weight basis)	1380 ± 28.4		
**Vitamins (mg/ 100 g on dry weight basis)**			
Vitamin B1	5.53 ± 0.9		
Vitamin B2	4.99 ± 0.78		
Vitamin B7	46 ± 4.5		
Vitamin B9	9.88 ± 1.23		
Vitamin E	8.98 ± 0.9		
**Phytopigments (mg/100 g on dry weight basis)**			
Β-carotene	1480 ± 10.2		
Chlorophylls	2350 ± 29.7		
Phycocyanin	52 ± 2.3		
**Microbiological quality (CFU g^−1^)**			
Total plate count	< 2 × 10^2^		
Total coliforms	< 10		
Yeasts and molds	Negative		
*Escherichia coli*	Negative		
*Salmonella* spp.	Negative		
*Listeria* spp.	Negative		
*Staphylococcus aureus*	Negative		
**Specific contaminants (ppm)**			
Arsenic	< 0.5		
Mercury	< 0.05		
Cadmium	< 0.2		
Lead	< 0.5		
Pesticides	Negative		
**DPPH radical-scavenging activity (%) at 100 µg mL^−1^**	42 ± 0.54		

**Table 2:** Effect of different treatments on body and reproductive organs weights in control and treated mice with bifenthrin (BF), SP, and their combination (SP + BF) ^a^.

Parameters	C	BF	SP + BF	SP
**Initial body weight (g)**	31.5 ± 3.00	30.25 ± 4.92	27 ± 1.00	27 ± 2.83
**Final body weight (g)**	35 ± 4.69	29.75 ± 3.30	30 ± 2.63	30.75 ± 4.27
**Body weight gain (g)**	3.5	−0.5 ^B^	3 ^E^	3.75 ^E^
**Absolute organs weights (g)**				
**Testes**	0.3 ± 0.09	0.17 ± 0.004 ^B^	0.23 ± 0.005 ^D^	0.24 ± 0.004 ^D^
**Epididymides**	0.12 ± 0.008	0.07 ± 0.016	0.08 ± 0.008	0.10 ± 0.012

^a^ Data are presented as mean + SEM (standard error mean). All groups vs. control group: ^B^ *p* < 0.01. All groups *vs.* BF group: ^D^ *p* < 0.05; ^E^ *p* < 0.01.

**Table 3:** Effect of different treatments on sperm parameters (cell density, motility, viability, and morphology) in control and treated mice with BF, SP, and their combination (SP + BF) ^a^.

Sperm parameters	Control	BF	SP + BF	SP
**Spermatozoa count per epididymis (×10^6^)**	5.82 ± 0.28	2.3 ± 2.45 ^C^	5.45 ± 0.82 ^F^	5.58 ± 0.91 ^F^
**Motility (%)**	76.75 ± 15.56	45 ± 7.07 ^B^	81.25 ± 7.5 ^E^	91.75 ± 7.89 ^F^
**Viability (%)**	97.5 ± 1.00	95 ± 2.45 ^A^	96.75 ± 2.06 ^D^	98 ± 0.82 ^D^
**Abnormal forms (%)**	5.75 ± 2.06	12.75 ± 2.87 ^B^	7.5 ± 0.02 ^E^	3.5 ± 1.00 ^F^

^a^ Data are presented as mean + SEM (standard error mean). All groups *vs.* control group: ^A^ *p* < 0.05; ^B^ *p* < 0.01; ^C^ *p* < 0.001. All groups *vs.* BF group: ^D^ *p* < 0.05; ^E^ *p* < 0.01, ^F^ *p* < 0.001.

## Supplementary Materials

The following are available online at https://www.mdpi.com/2218-273X/10/5/753/s1, Table S1: Sequences of the primers used in this study.
