# Supplementary material for: Protective Role of Spirulina platensis against Bifenthrin-Induced Reprotoxicity in Adult Male Mice by Reversing Expression of Altered Histological, Biochemical, and Molecular Markers Including MicroRNAs
Source: Biomolecules. 2020 May 12;10(5):753. doi: 10.3390/biom10050753 (PMC7277961; doi:10.3390/biom10050753)
Supplement: Supplementary file 1 [file biomolecules-10-00753-s001.pdf]

Table S1 Sequences of the primers used in this study.

Gene	Forward Sequence	Reverse sequence
**U6 snRNA**	5'- CGCAAGGATGACACGCAAATTC-3'	
**miR-34c**	5'- GCAGTGTAGTTAGCTGATTGC -3'	
**miR-17**	5'-AAAGTGCTTACAGTGCAGGTAG-3'	
**miR-122**	5'-TGGAGTGTGACAATGGTGTTTG-3'	
**miR-34b-5p**	5'-AGGCAGTGTAATTAGCTGATTGT-3'	
**miR-34b-3p**	5'-AATCACTAACTCCACTGCCATC-3'	
**miR-449a**	5'-TGGCAGTGTATTGTTAGCTGGT-3'	
**miR-449c**	5'-GCAGTGCATTGCTAGCTGG-3'	
**miR-509**	5'-GTACTCCAGAATGTGGCAATC-A-3'	
**miR-146b**	5'-GTGAGAACTGAATTCCATAGG-C-3'	
**actin**	5'-ACTGTCGAGTCGCGTCCACC-3'	5'-GGGAATACAGCCCGGGGAGC-3'
**P53**	5'-AAGAGAGCGCTGCCCACCTG-3'	5'-TACGCCCGCGGATCTTGAGG-3'
**Bid**	5′-CGA AGA CGA GCT GCA GAC-3′	5′-CTC GTT TCT AAC CAA GTT CC-3′
**Bcl2**	5'-CATGTGGCTATGCGGCAATG-3'	5'-TCTCGGAGGGGAGGATTTCA-3'
**ApafI**	5'-AAGGATGGAAAGTCTGTGT-3'	5'-CTCTAGATGAAGCCATGTCG-3'
**Fas Ig**	5'-TGCATTGACGGCCGGGTCAT-3'	5'-CAGCGCCACGGTTCCTGTCT-3'
**Bax**	5'CCGCACGTCCACGATCAGTCA-3'	5'-ATCACTGCCGCTGCCTCTCG-3'
**Cytochrome c**	5'-GAGGCAAAGCATAAGACT-3'	5'-TACTCCATCAGGGTATCCTC-3'
**TNFα**	5'- GTCTACTGAACTTCGGGGTGAT-3'	5'-GGCTACAGGCTTGTCACTCG-3'
**Caspase9**	5'-AGTTCCCGGGTGCTGTCTAT-3'	5'-GCCATGGTCTTTCTGCTCAC-3'
**Caspase 3**	5'-GCGGGGAGCTTGGAACGCTAA-3'	5'-CGTCCACATCCGTACCAGAGCG-3'
**Caspase 8**	5'-TGCCCTCAAGTTCCTGTGCTTGGAC-3'	5'-GGATGCTAAGAATGTCATCTCC-3'
**P45017-α**	5'-CCATCCCGAAGGACACACAT-3'	5'-GTGGCTGGTCCCATTCATTT-3'
**P450scc**	5'-CCATCAGATGCAGAGTTTCCAA-3'	5'-TGAGAAGAGTATCGACGCATCCT-3'
**SRB1**	5'-CCCTTCGTGCATTTTCTCAAC-3'	5'-CATCCCAACAAACAGGCCA-3'
**PBR**	5'-AGTTCGTGGCACTGCATAAGC-3'	5'-GCTGCCCATTCTCTCCTCCTA-3'
**StaR**	5'-TCGTGAGCGTGCGCTGTACC-3'	5'-GACACCACTCTGTGCTCCGGCCA-3
**LDL-R**	5'-GGAAAATGCATCGCTAGCAAGT-3'	5'-ATTGGACTGACAGGTGACAGACA-3'
**17β-HSD**	5'-TGACCAAGACCGCCGATGAGT-3'	5'-CATGAGCAAGGCAGCCACAGG-3'
**3β-HSD**	5'-GCTGCTGCACAGCCCTCCTAA-3'	5'-ACCCTCCTGCTCCTGTCACCA-3'
